# Comprehensive Single Molecule View of Transcriptional Dynamics in Development

**DOI:** 10.1101/2025.10.28.685222

**Published:** 2025-10-29

**Authors:** Thomas W Tullius, Tohn Borjigin, Michael Levine

**Affiliations:** Lewis-Sigler Institute, Department of Molecular Biology, Princeton University, Princeton, NJ 08544

## Abstract

Dynamic patterns of gene expression drive animal development. These transcriptional dynamics depend on the interplay of diverse developmental enhancers within complex regulatory landscapes. Here, we employ a single-molecule genomic method, Fiber-Seq, to capture the simultaneous transcriptional and regulatory states of millions of 20–30 kb chromatin fibers in the 2–4hr *Drosophila* embryo. These snapshots of genes and their surrounding regulatory context reveal that the basic unit of transcription at highly transcribed genes is a convoy of 2–14 tightly spaced polymerases (Pol II). Further, a subset of templates exhibits hyperbursting, whereby most or all nucleosomes are evicted from the gene body facilitating maximum rates of transcription. We demonstrate that hyperbursting is achieved through cooperation of numerous cis-regulatory elements, and that the resulting nucleosome eviction appears to trigger a novel silencer in the *ftz* 3’ UTR. We anticipate that similar mechanisms are used by vertebrate processes requiring intense transcription like somitogenesis and erythropoiesis.

## Introduction

Most previous studies of gene regulation in development focus on interactions of individual enhancers with their target promoters. There are numerous examples, including long-range regulation of the Sonic Hedgehog (*Shh*) gene by the distal ZRS enhancer in developing limb buds of mammalian embryos (1). However, developmental control genes such as *Shh* contain complex regulatory landscapes containing numerous enhancers (2).

There are several documented examples of cis-regulatory interactions influencing the timing of gene expression, such as limb patterning by the Hoxd locus in mice (3). There are also examples in the *Drosophila* embryo, including enhancer switching in the Brinker locus (4) and the timing of segmentation stripes of even-skipped expression (5). These examples of co-dependencies of cis-regulatory elements in development are anecdotal as they depend on painstaking functional analyses in transgenic embryos. Here, we employ a high-throughput single-molecule method, Fiber-Seq (6,7), to examine the relationship of enhancer activities and transcriptional states for hundreds of developmental control genes on millions of individual chromatin fibers in the early *Drosophila* embryo.

Computational analyses of these comprehensive datasets identified “hyperbursts”, an extreme state of transcriptional activation whereby convoys of 2–14 tightly spaced Pol II complexes evict most or all nucleosomes across gene bodies. Hyperbursts depend on the cooperation of multiple cis-regulatory elements, including those dedicated to transcriptional bursting. A newly identified silencer element in the *ftz* 3’ UTR appears to be triggered by hyperbursts and nucleosome depletion. This element is the source of an anti-sense RNA that is expressed in regions of the embryo where *ftz* is known to be repressed (5). We discuss the implications of these findings with respect to mammalian developmental and disease processes requiring intense periods of transcription such as somitogenesis (8) and erythropoiesis (9).

## Results

**Figure 1:**
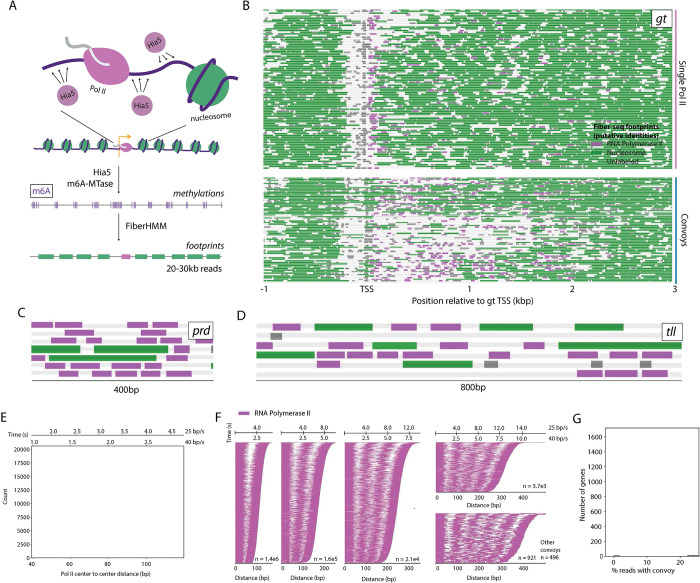
Transcription at highly expressed genes is driven by Pol II convoys A. Schematic of Fiber-seq and FiberHMM. B. Example Fiber-seq reads at the *gt* locus. Reads are split between reads with (top) single and (bottom) convoys of Pol II footprints. C. Seven Fiber-seq reads at the *prd* locus showing Pol II convoys. D. Six Fiber-seq reads at the *tll* locus showing Pol II convoys. E. Histogram of the basepair distance between convoy Pol II footprints. A pair of secondary x-axes above estimate time from elongation rates. F. Pol II footprints within different size convoys, aligned to the 5’ end of the first Pol II footprint. Secondary x-axes above estimate time from elongation rates. Plots show 500 sampled fibers. G. Histogram of percent reads with a Pol II convoy across all expressed genes (n=4329).

We employed a high throughput single molecule method, Fiber-Seq, to explore the extreme transcriptional dynamics seen in the early *Drosophila* embryo (Fig. 1A). Our efforts centered on 2–4 hr embryos (nuclear cycle, NC, 13, 14, and early gastrulation) since this is the time when the fate map of the adult fly is established through the regulation of over 100 patterning genes by several hundred developmental enhancers (10,11,12). We obtained ~4000-fold coverage of the entire genome with single molecule read lengths averaging ~23 kb. This coverage is among the highest of single molecule genomic datasets ever produced.

FiberHMM was used to visualize the chromatin-bound proteome of every individual fiber (7). We incorporated previously published PRO-Seq assays (13,14) to facilitate the identification of different transcriptional states, including Pol II pausing and PIC occupancy (7,15,16) (Fig. S1A,B), as well as Pol II footprints that extend beyond the 3’ UTR (Fig. S1C). Numerous Pol II footprints were also identified within gene bodies correlating with PRO-seq elongation signals (Fig. 1B). There is a strong correspondence between the fraction of fibers showing Pol II occupancy at a given gene and the overall fraction of cells expressing that gene in scRNA-seq from NC14 embryos (Figure S1D-H) (17).

### Pol II convoys at highly transcribed genes

The patterns of elongating Pol II footprints revealed an unexpected mode of transcription enriched at highly transcribed developmental patterning genes. Chromatin fibers exhibiting low or moderate levels of transcription possess individual elongating Pol II footprints sporadically distributed across the gene body. By contrast, strongly transcribed fibers contain “convoys” of tightly spaced Pol II footprints (Fig. 1B-D, Fig. S1I-K). Within these convoys, polymerases were spaced an average of 60 bp (center-to-center), leaving only 5–10 bp between footprints, far tighter than typical internucleosomal spacing (Fig. 1E, Fig. S1J). This minimal spacing corresponds to the release of Pol II every 2–3 seconds (18,19), approaching the theoretical limit imposed by polymerase sterics (Fig. 1E). These release rates are consistent with the large Pol I convoys seen at rRNA genes (Fig. S1M; 20) and previous studies of Pol II release (21,19).

Convoys most frequently consist of 2–4 polymerases but rarely extend up to 14, representing continuous Pol II release events for upwards of 30 seconds (Fig. 1F). Strikingly, while these convoys are prevalent at highly transcribed developmental patterning genes, they are also observed for an average of ~7% of all chromatin fibers at “typical” genes, suggesting a general mechanism for the release of small Pol II clusters during intense periods of transcription (Fig. 1G, Fig. S1N).

### Hyperbursts evict nucleosomes

**Figure 2:**
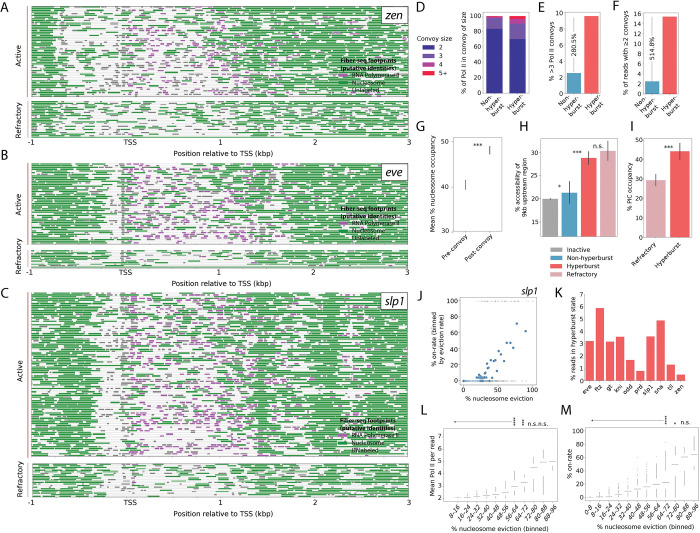
Hyperbursts evict nucleosomes from gene bodies A. Fiber-seq reads at *zen* with >50% nucleosome depletion split by active (Top) and refractory (Bottom) fibers. B. Fiber-seq reads at *eve* with >50% nucleosome depletion split by active (Top) and refractory (Bottom) fibers. C. Fiber-seq reads at *slp1* with >50% nucleosome depletion split by active (Top) and refractory (Bottom) fibers. D. Percentage of Pol II footprints in different convoy sizes in non- and hyperburst fibers. E. Percentage of larger than 3 Pol II convoys in non- and hyperburst fibers. F. Percentage of fibers with more than 1 Pol II convoy in non- and hyperburst fibers. G. Nucleosome occupancy 300 bp upstream (excluding promoter) and downstream of convoys for the 100 most hyperburst-enriched genes. Error bars, 95% CI from 1000× bootstrap; ***p < 10^−4^ (rank-sum) H. Percent accessibility of the 10kb upstream of TSSs on non- and hyperburst fibers. Errorbars indicate 95% confidence interval from 1000x bootstrapped resamplings. Significance is indicated between bars; ***: p < 10^−4^, * p < 10^−2^, n.s. p>.05 (rank-sum). I. Percent PIC occupancy of non- and hyperburst fibers. Errorbars indicate 95% confidence interval from 1000x bootstrapped resamplings. Significance is indicated between bars; ***: p < 10^−4^ (rank-sum). J. Scatterplot of on-rate (% reads with ≥1 convoy) of fibers binned by centiles of percent nucleosome eviction at *slp1*. Grey points are individual active or inactive fibers. K. Percentage of hyperburst reads for ten patterning genes with the highest levels of hyperbursting. L. Mean Pol II footprints per read for 100 most hyperburst-enriched genes, binned by nucleosome eviction; ***p < 10^−4^, **p < 10^−3^, n.s. > 0.05 (Mann-Whitney U). M. On-rate for 100 most hyperburst-enriched genes, binned by nucleosome eviction; ***p < 10^−4^, **p < 10^−3^, n.s. > 0.05 (Mann-Whitney U).

Individual Pol II footprints on fibers with low to moderate transcription exhibit a regular spacing periodicity of ~147 bp, being found within internucleosomal linker regions (Fig. S2A). By contrast, Pol II convoys found at highly transcribed genes appear to transiently displace nucleosomes in their path (Fig. 1B, S2A), with a single nucleosome evicted for every ~2.5 Pol II footprints within a convoy (Fig. S2B). Most dramatically, 0.5% to 7% of the fibers comprising 285 highly active genes display “hyperbursting”, a state of intense transcription whereby chromatin fibers lack at least 50% of the nucleosomes normally distributed across the gene body (Fig. 2A-C). Compared to fibers without nucleosome eviction, hyperburst fibers contain a 3-fold average increase in the number of Pol II convoys consisting of 3 or more polymerases and a 5-fold average increase in the number of fibers containing at least two Pol II convoys within the gene body (Fig. 2D-F, Fig. S2C-E). Developmental patterning genes are strongly enriched among the genes with the highest level of hyperbursting, including all 5 genes with the highest fraction of hyperburst reads: *ftz, sna, gt, tll,* and *eve*. Consistent with a transcription-driven mechanism for nucleosome eviction we note that nucleosome densities are lower upstream of Pol II convoys than downstream (Fig. 2G).

A subset of fibers exhibiting nucleosome depletion lack Pol II footprints (Fig. 2A-C). We believe that these “refractory fibers” represent off periods between transcriptional bursts. Several arguments support this view. In addition to exhibiting extensive nucleosome depletion, putative refractory fibers possess upstream cis-regulatory element (CRE) accessibility patterns that are far more similar to those seen for hyperburst fibers than weakly transcribed fibers (Fig. 2H, Fig. S2F). Further, these refractory fibers show a notable depletion of PIC footprints, which is consistent with a transcriptional “off” state (Fig. 2I).

The ratio of active to refractory hyperburst fibers provided an opportunity to capture the transcriptional kinetics of intensely active developmental genes during periods of maximum transcription (Fig. 2J, S2G). Across all fibers, Fiber-seq derived estimates of on-rates are similar to those seen across all nuclei for live-imaging of *ftz* transcription (5), with estimates of 14.4% and 16%, respectively. While the fraction of fibers in the hyperburst state varied widely from gene to gene (Fig. 2K), the on-rates and amplitude of transcription plateaued at high levels of nucleosome eviction (Fig. 2L,M). At >80% eviction hyperbursting genes reached an average on-rates of ~68%, whereas weak or moderately active fibers exhibiting less than 40% eviction possess on-rates of just ~5% (Fig. 2L,M). This constancy of hyperactive on-rates suggest that developmental patterning genes reach comparable states of maximal transcription and is consistent with the two-state model for transcriptional bursting based on MS2/PP7 live-imaging methods (22).

### Dynamic CREs for different states of transcription

**Figure 3:**
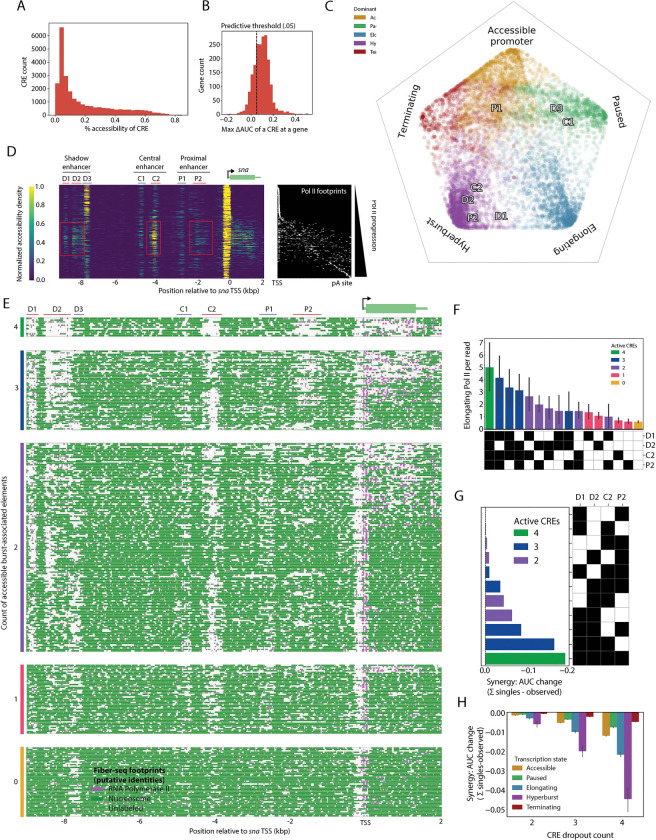
Hyperburst-associated regulatory elements function cooperatively at *sna* A. Percent accessibility for FIRE-identified CREs <9kb upstream of an expressed gene. B. Maximum ΔAUC for an individual CRE upstream of each expressed gene. C. Simplex plot showing regression coefficients predicting transcriptional states (vertices) from CRE accessibility (points); colors indicate strongest coefficient. D. Fiber-seq reads at *sna* colored by local accessibility density. CREs schematized above; red boxes, active hyperburst-associated CREs. Right, same reads with Pol II footprints in white. E. Fiber-seq at sna grouped by number of accessible hyperburst-associated CREs (count indicated on left); 50 sampled reads shown for 0–1 active elements. F. Count of elongating Pol II footprints per read for combinations of hyperburst-associated CREs, sorted by mean count. Combinations of elements are schematized below, with black boxes showing accessibility. Bars are colored by accessible element counts. Errorbars represent the 95% confidence interval from 1000x bootstrapped resamplings of the fibers. G. Synergy scores from logistic-dropout models showing cooperative effects of *sna* hyperburst-associated CRE combinations (difference between sum of single-dropouts and combination dropout on model AUC). H. Synergy scores from logistic-dropout models showing cooperative effects of CRE combinations (difference between sum of single-dropouts and combination dropout on model AUC).

Hyperburst fibers exhibit enhanced accessibility of upstream regulatory regions as compared with those fibers displaying moderate or low levels of transcription, suggesting coordinated remodeling of regulatory landscapes to achieve maximum transcription (Fig. 2G). To test if hyperbursting harnesses unique regulatory mechanisms, we employed Fiber-Seq Inference of Regulatory Elements (FIRE) to identify putative *cis* regulatory elements (CREs) within the first 10 kb upstream of all TSSs (23). Known and putative CREs exhibit highly variable accessibility from fiber to fiber (Fig. 3A), illustrating a dynamic regulatory landscape driven by extensive chromatin remodeling.

We then tested the relationship between CRE accessibility and transcription by directly associating dynamic accessibility patterns with different transcriptional states on the same individual chromatin fibers, training logistic regression models with activities of CREs as predictors and transcriptional state assignments as responses. Transcriptional states were chosen to capture distinct stages of gene activation: poised (accessible promoter and paused Pol II), active transcription (non-hyperburst elongation and hyperburst), and attenuated (Pol II footprints only within the 3’ termination region). Overall, 73% of genes had at least one CRE that was predictive of a transcriptional state, and for 68% of genes, the overall pattern of CRE activity was predictive of at least two different transcriptional states (Fig. 3B, Fig. S3A). These observations support specialized roles for individual CREs in different phases of the transcription cycle (Fig. 3C, Fig. S3B,C). Other than poised promoters, combined CRE activities were particularly predictive of hyperbursts (Fig. S3D-I).

### Hyperburst regulatory elements at the snail locus

To explore the regulatory basis for hyperbursts we turned to *snail* (*sna*), which encodes a key determinant of epithelial-mesenchyme transitions (EMT) including mesoderm invagination at the onset of gastrulation (24, 25). Classical studies identified two developmental enhancers, a distal, “shadow enhancer” (herein D) which is the primary activator, and a proximal enhancer (herein P) that has a weak activating effect and a potential role in attenuation (26, 27, 28). FIRE identified seven distinct modules in the *sna* 5’ regulatory region, including three modules in the D enhancer and another two in P. A third, bipartite region was also identified in central regions (C1 and C2) that was not previously implicated in *sna* regulation. All seven modules appear to possess specialized functions with respect to states of transcription: hyperburst-associated (D1, D2, C2, P2), pause-associated (D3, C1), and attenuation-associated (P1) elements (Fig. 3C,D). This combinatorial logic suggests inter-connected regulation that transcends independent action of separate enhancer modules.

These putative redundancies could be explained either by overlapping activities or distinct contributions of different modules. Consistent with the former model, the burst-associated modules (D1, D2, C2, P2) frequently showed co-accessibility: 30% of fibers had one module active while 17% had two or more (Fig. 3E). Pol II footprint counts and gene-body accessibility scaled with the number of active modules (Fig. 3F, Fig. S3J). This scaling was more than additive, with the overall contributions of multiple elements (in particular, D1, D2, and C2) being higher than expected when dropped out of regression training, supporting a model wherein CREs cooperatively tune transcriptional output, rather than functioning redundantly and separately (Fig. 3G). This cooperativity is broadly detected, with hyperburst-associated elements generally showing greater synergy as compared with CREs associated with pausing, elongation, or termination (Fig. 3H, S3K).

### Developmental trajectories at the *ftz* locus

**Figure 4:**
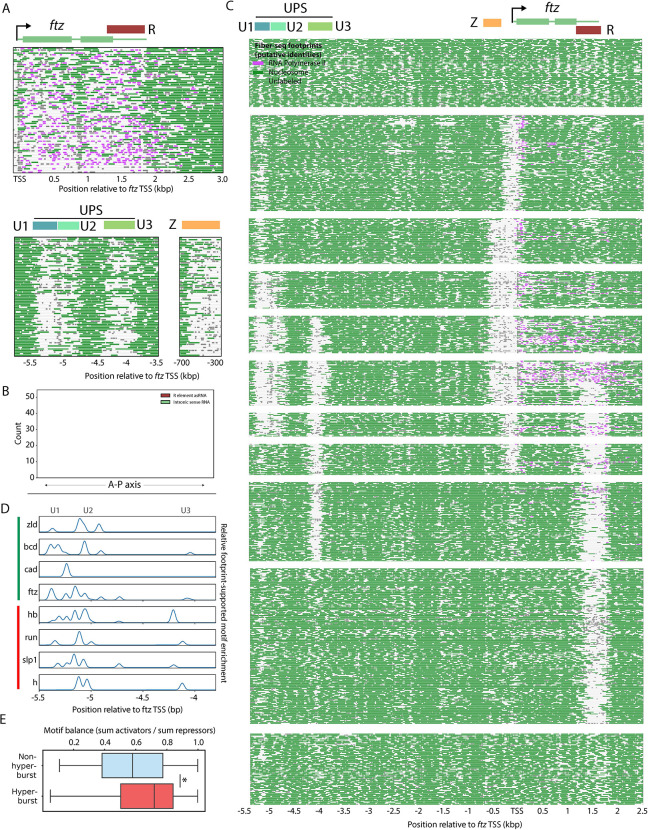
Hyperburst-associated regulatory elements attenuate *ftz* expression A. (Top) Hyperburst fiber-seq reads at the *ftz* locus. (Bottom) The same Fiber-seq reads, ordered identically but (Left) at the UPS and (Right) at the Zebra element. B. Density of nuclei expressing *ftz* sense intronic RNA and *ftz* R asRNA across the anterior-posterior axis measured via smFISH (R-element: n = 356, intron: n = 663). C. Fiber-seq reads overlapping the *ftz* locus and regulatory region, divided into steps based on the top-scoring trajectory of transcriptional activation and attenuation. Reads with no accessible elements are sampled to 150 total. D. Enrichment of activator and repressor motifs at the UPS, with each CRE module indicated via colored blocks. Motifs are identified with a permissive log-odds threshold (≥ 2.0) and filtered by overlapping subnucleosomal Fiber-seq footprints in at least 5% of total fibers. E. Boxplot showing the ratio of activator and repressor motif enrichment for non- and hyperburst-associated CREs. Motifs are identified with a permissive log-odds threshold (≥ 2.0) and filtered by overlapping subnucleosomal Fiber-seq footprints in at least 5% of total fibers.

We next sought to determine the role of hyperbursting within the developmental trajectory of a key pair-rule gene, *ftz*, which exhibits remarkable transcriptional dynamics during NC14 (29). Live-imaging assays reveal activation, bursting, attenuation, and silencing within a span of just one hour (5). Classical gene fusion studies identified two developmental enhancers for ftz regulation, a proximal Zebra element (Z) located adjacent to the promoter and a distal autoregulatory element (UPS) located ~5 kb upstream (30, 31, 32, 33). Z initiates *ftz* expression in early embryos, while the UPS maintains and refines these stripes in older embryos (29). As seen for *sna*, *ftz* hyperburst fibers contain maximally accessible CRE modules, in this case the Z enhancer and UPS (Fig. 4A).

FIRE revealed unexpected complexity within the UPS, having identified three distinct modules (U1, U2, U3), each associated with distinct transcriptional states (Fig. S4A). U1 is associated with poised, elongating, and hyperbursting states, whereas U2 is an extension of U1 and is associated primarily with hyperbursting. U3 is associated with both hyperbursting and attenuation (Fig. S4A). This analysis also identified a novel CRE, “R”, that is associated both with hyperbursting and attenuation (Fig. 4A, S4A,B). In support of a role as an attenuator, the R element produces an antisense RNA that is expressed in interstripe regions where *ftz* is repressed during stripe refinement (Fig. 4B, S4C,D). R is also enriched for autoregulatory and repressive binding motifs (Fig. S4E).

Only 17 of the 32 possible accessibility combinations of these elements were observed in more than 1% of fibers, indicating a constrained regulatory logic. The arrangement of *ftz* fibers that we present is based on the highest scoring route from a graph-based trajectory analysis, where nodes represent the observed combinations of regulatory states and edges represent single-element transitions prioritizing those supported by the maximum number of fibers (Fig. 4C, Fig. S4F,G). The primary activation trajectory proceeded through six ordered stages: **(i) Promoter opening** (102 fibers), the promoter becomes accessible while enhancers remain closed; **(ii) Early activation** (110 fibers), Zebra or U1 become accessible and initiate low levels of transcription; (iii) **Burst amplification** (62 fibers), U2 and U3 modules open and transcription intensifies; (iv) **Hyperbursting** (46 fibers), R element opens during maximal transcription; (v) **Activation shutdown** (67 fibers), Zebra/U1/U2 close while low levels of transcription continue; and (vi) **Active repression** (230 fibers), promoter closes but U3/R remain accessible (Fig. 4C, Fig. S4F,G).

This trajectory aligns with known developmental timing, with the Z element initially activating low and moderate transcription, followed by UPS activation via autoregulation leading to sustained, bursty transcription (29). As observed for *sna*, hyperbursting is associated with widespread accessibility of the regulatory landscape, particularly the specialized bursting elements (e.g., U2 and U3). This extensive remodeling of the regulatory landscape and *ftz* gene body exposes repressor motifs (e.g., Hb, Slp1, Run, and Hairy) across the U2, U3 and R elements (Fig. 4E, Fig. S4E). This observation is consistent with a role for hyperbursting in sensitizing *ftz* to repressive regulatory inputs. Globally, the ratio of repressive and activating motifs within hyperburst-associated CREs is more balanced than the motif distributions seen for CREs associated with other states such as pausing and termination. These observations suggest that bifunctional activation and attenuation may be widespread (Fig. 4E; see below).

## Discussion

We have presented evidence that many or most developmental control genes display hyperbursting, a short-lived peak of transcriptional activity coinciding with extensive eviction of nucleosomes across gene bodies. A similar mass eviction was previously reported for hsp70 upon heat shock in *Drosophila*, where denuded chromatin fibers facilitate peak rates of transcription (34). The developmental control genes we characterized appear to achieve similar peaks of activity, consistent with previous live-imaging studies suggesting similar on-rates for *Drosophila* gap genes (35). This suggests that eviction of nucleosomes may be a general mechanism to achieve maximized transcription, and we expect that this state will also be observed in mammalian systems requiring extreme transcriptional outputs like erythropoiesis and somitogenesis.

Our analyses connecting CRE accessibility to transcriptional states suggest that classically defined developmental enhancers are often composed of multiple elements with distinct functions. Many of these elements are only accessible on fewer than 10% of chromatin fibers, yet their functional importance is supported by strong associations with specific transcriptional states. This observation supports an outsize role for rapid chromatin remodeling in mediating developmental enhancer switching and fine-tuning gene expression. For example, the full-length distal *sna* (aka shadow) enhancer appears to contain three distinct cis-regulatory elements (D1, D2, and D3) with differing activities. D3 accessibility coincides with paused Pol II at the *sna* promoter, whereas D1 and D2 accessibility coincides with Pol II elongation and hyperbursts, respectively. These distinct activities could explain why the distal *sna* enhancer directs especially robust expression of reporter genes in transgenic embryos (36).

An interesting implication of this analysis is the apparent necessity of long-range cooperation of numerous regulatory elements to achieve hyperbursting of developmental control genes. Hyperbursting of *sna* does not depend solely on the D1 and D2 elements within the distal enhancer but also appears to require maximal accessibility of the C2 and P2 elements located 3 kb and 6 kb downstream of D2, respectively. Similarly, hyperbusting of *ftz* coincides with peak accessibility of all three elements comprising the autoregulatory UPS enhancer as well as the proximal zebra enhancer, separated by over 4 kb.

The extensive regulatory accessibility associated with hyperbursting suggests two mechanisms for triggering auto-attenuation. First, many hyperburst-associated regulatory modules comprising developmental enhancers appear to possess dual activator and repressor activities. For example, the U1 element of the *ftz* UPS autoregulatory enhancer achieves maximum accessibility, along with the proximal zebra enhancer, as transcription increases. U1 contains known activator binding motifs (e.g., Bicoid and Caudal), but these are not sufficient for high levels of transcription. Hyperbursts are observed only when the neighboring U2 and U3 modules also become accessible, possibly through spreading of U1 activation. U2 and U3 are enriched for both autoregulatory activator (e.g., Ftz) and repressor binding motifs (Hairy and Runt), supporting a function both in enhancing hyperbursts and subsequent attenuation (Fig. 4D).

The second potential mechanism of attenuation is more direct. Namely, the *ftz* R element appears to become accessible only upon hyperbursting-driven nucleosome eviction in the 3’ UTR (Fig. S4E). The R element also appears to mediate repression at later stages of development when *ftz* is regulated by a new set of enhancers during neurogenesis (Fig. S4H) (Heffer et al., 2013). It is unclear whether antisense transcription arising from the R element directly contributes to the attenuation of *ftz* expression in interstripe regions. It is possible that antisense transcription precludes transcription of sense RNAs. Alternatively, antisense RNAs might work post-transcriptionally to degrade lingering *ftz* mRNAs produced in interstripe regions at earlier stages of development when *ftz* is preferentially transcribed in future interstripes (Fig. S1I) (5).

Our Fiber-seq analysis provides a glimpse into the inter-connected activities of the complex regulatory landscapes controlling gene activity during development. This analysis suggests a level of regulatory integration and cooperativity not envisioned by the traditional modular view of the regulatory genome, whereby separate enhancers function in an autonomous manner to produce composite patterns of gene expression. Overall, enhancers and their constituent modules are rarely active as individual entities, but rather, many transcriptional states (most notably hyperbursts) appear to require the cooperation of multiple modules. Recent studies provide some evidence for regulatory integration, including examples of long-range enhancer-promoter compatibility across neighboring TADs (37, 38). We expect that further investigation of regulatory networks at the single-fiber scale will reveal that this cooperativity is a general feature of the gene activities controlling dynamic developmental processes across the metazoa.

## Methods

### Fiber-seq

2–4hr and 6–8hr embryos were collected and dechorionated for 1 minute 45 seconds in 50% bleach. Embryos were then resuspended in homogenization buffer (250mM Sucrose,10mM Tris-CL, pH 8.0, 25mM KCl, 5mM MgCl_2_, 0.1 mM, 0.1% Triton X-100) and dounced 40 times using a tight dounce to isolate nuclei. Nuclei were filtered via a 40um filter and spun down at 350 × g for at 4°C for 10 minutes. Pellets were resuspended in 30 μL Buffer A (15 mM Tris-Cl pH 8.0, 15 mM NaCl, 60mM KCl, 1 mM EDTA, 0.5 mM EGTA, 0.5 mM Spermidine). 0.8 mM SAM and 200 U of Hia5 were then added, followed by incubation for 10 minutes at 25°C. The reaction was quenched with the addition of 1% final SDS and mixing using a wide-bore pipette tip. Samples for several collections were pooled, and their DNA was purified using the Promega Wizard HMW DNA Extraction Kit (Promega A2920) and submitted for library preparation and sequencing. A total of 24 collections for each timepoint were pooled, barcoded, and sequenced on the Pacific Biosciences Revio. 11 total sequencing runs were carried out to achieve around ~4000x coverage of the genome.

### FiberHMM

Footprints were called on Fiber-seq reads using FiberHMM. FiberHMM is based on a hidden Markov model (HMM) with two hidden states-- accessible and inaccessible. To account for sequence-related biases from Hia5 or the methylation caller, at each position the model takes into account both the base and its surrounding 6 bp sequence context. The emission probabilities used in the model are the probabilities of methylation of a given base with its +/− 3 bp sequence context in an accessible or inaccessible state based on experimental-derived methylation rates from control datasets. The probability of methylation given an accessible state was based on the methylation frequency in a dataset generated from dechromatinized *Drosophila* S2 cell genomic DNA. The probability of methylation given an inaccessible state was based on the methylation frequency in a dataset generated from *Drosophila* S2 cells untreated with Hia5. Transition and starting probabilities for the HMM were carried over from a model trained on *Drosophila* S2 genomic DNA with initial probabilities picked from the Dirichlet distribution with all parameters set to 1 (7).

### Defining accessibility and footprints

Nucleosome footprints were in general defined as footprints larger than 90bp. Pol II footprints were defined as footprints between 40 and 60 bp, overlapping signal from PRO-seq. PIC footprints were defined as either 20–40bp overlapping a TATA box (TBP), or 60–80 bp overlapping the TSS, as defined via CAGE-seq (40). Transcription factor footprints were defined as <90bp, not overlapping PRO-seq signal. To account for transcription factor footprints in close proximity leading to merged footprints, footprints larger than 90bp and less than 200bp were called as transcription factors if there was support from at least 50 reads with subnucleosomal footprints that perfectly aligned with both ends of the larger footprint. Accessible regions in general were defined as all gaps between nucleosomal footprints, ignoring called transcription factor and polymerase footprints.

### Comparing Fiber-seq to PRO-seq, scRNA-seq, and *in situ* hybridizations

Processed PRO-seq and scRNA-seq datasets from nuclear cycle 14 were retrieved from GSE211220 and DVEX respectively. For PRO-seq, genes were binned into equal deciles of signal within the pause site (TSS to +200bp), body (+200bp to end), or termination region (end + 500bp). Mean fiber-seq footprint occupancy (pause, PIC, elongating, terminating) within the same regions was then calculated for each gene, with the pooled means plotted for each decile. For comparisons to scRNA-seq, per-cell matrices were accessed and the count of genes with at least one cell showing one transcript was calculated. This was then compared to the fraction of reads showing an accessible promoter or the fraction of reads showing any Pol II footprint within any of the above regions was plotted. Gene expression patterns for comparison to accessibility in Figure S1 were retrieved from the BDGP in situ hybridization database (Berkeley *Drosophila* Genome Project, https://insitu.fruitfly.org; accessed 27 Oct 2025).

### Subsets of genes

For analyses involving all genes, active genes from scRNA-seq as defined above (n=4329) were used in subsequent analyses. For analyses involving a subset of developmental genes, this curated list of 63 NC14 developmental patterning genes were used: bcd, cad, hb, Kr, kni, gt, tll, hkb, eve, run, ftz, odd, prd, slp1, slp2, opa, en, inv, wg, hh, ptc, smo, ci, dl, twi, sna, rho, sim, vnd, ind, msh, tor, trk, tsl, Egfr, spi, vn, dpp, scw, sog, tld, tsg, tkv, sax, put, Mad, Medea, brk, shn, pan, zen, lab, pb, Dfd, Scr, Antp, Ubx, abd-A, and abd-B.

### Defining convoys

Convoys were defined as groups of two or more Pol II footprints within a gene body separated by 100bp or less (approximately two Pol II footprints maximum). This definition was picked based on calibration curves shown in Figure S1I, which found that a maximum 100bp separation captured 95% of grouped polymerases.

### Transcription state definitions

Transcription states were defined as follows for regression analyses. An accessible promoter was defined as at least 100bp of accessibility on average surrounding the TSS of a gene on a read. A paused Pol II was defined as mean footprint size of 40–60 bp within the first 50bp downstream of the TSS. Elongating Pol II was defined as at least one called Pol II footprint found within the gene body. The hyperburst state was defined as at least 50% accessibility of the gene body.

### Defining on-rate, amplitude

On-rate was estimated as the fraction of fibers with at least one convoy of Pol II. Relative burst amplitude was estimated as the mean count of polymerases found on a fiber.

### Defining hyperbursts, active and refractory

Globally, hyperbursts were defined as fibers with less than 50% nucleosome occupancy within the first 1.5kb of the gene body (or less, if the gene was shorter, with all derivative calculations accounting for the shorter length). Refractory states were defined as fibers with that level of nucleosome eviction, but with no convoys of polymerase, while active states were defined as the population of fibers with convoys.

### Comparison of on-rates between Fiber-seq and live-imaging

On-rate for live imaging data for *ftz* was calculated using 181 background-normalized fluorescence measurements for 1541 cells taken at 15s intervals (5). A gaussian mixture model was applied to identify a threshold for a 99% confident active state, and the total count of active and inactive states were counted. For Fiber-seq, the fraction of reads at the *ftz* locus showing convoy activity was calculated and compared to the live-imaging result.

### Fiber-seq Inference of Regulatory Elements (FIRE)

FIRE was applied to the datasets using fibertools v0.7.0 (41). Aggregate traces of FIRE signal were then generated, with peaks called using scipy peak calling (minimum width = 20 bp, minimum height = 1/5^th^ of the maximum peak in the window). Peaks less than 100bp were expanded to 100bp, and overlapping peaks were merged. Accessibility of elements within these regions called based on footprinting as described above.

### Transcription state definitions

Transcription states were defined as follows for regression analyses. An accessible promoter was defined as at least 100bp of accessibility on average surrounding the TSS of a gene on a read with no polymerase footprints overlapping the gene. A paused Pol II was defined as a mean footprint size of 40–60 bp within the first 50bp downstream of the TSS with no other polymerase footprints. Elongating Pol II was defined as at least one Pol II footprint found within the gene body. The hyperburst state was defined as at least 50% accessibility of the gene body. Termination was defined as at least one Pol II footprint found within the 500bp after the annotated gene end.

### Logistic Regression Modeling of Transcriptional States

The probability that an individual chromatin fiber belonged to a specific transcriptional state—accessible promoter, paused, elongating, hyperbursting, or terminating—was modeled as a function of the accessibility of nearby cis-regulatory elements (CREs) identified by Fiber-seq Inference of Regulatory Elements (FIRE). For each gene, all putative CREs located within a 9 kb window upstream of the annotated transcription start site (TSS) were identified using FIRE. Accessibility was encoded on a per-read basis. The resulting binary feature matrix (reads × features) was constructed for each gene. Columns corresponding to transcriptional states (accessible, pause, elongating, hyperburst, terminating) served as response variables, and all remaining binary features were used as predictors.

Each transcriptional state was modeled independently for every gene using penalized logistic regression of the form:

$$logit\left(P\left(y_{i}=1\right)\right)=\beta_{0}+\Sigma \beta_{j}x_{ij}$$

where $y_{i}$ denotes the presence of the transcriptional state on read i and $x_{ij}$ denotes the accessibility of feature j on that read. Models were fit using the liblinear solver in scikit-learn (LogisticRegression) with an L1 penalty (C = 1.0, class_weight = ‘balanced’, max_iter = 500). L1 regularization encouraged sparsity and yielded interpretable coefficients indicating the direction and magnitude of association between each CRE and the transcriptional state. In addition to these **multi-feature models**, parallel **single-feature regressions** were fit in which each CRE was considered individually against the same response variable. The single-CRE fits provided baseline predictive contributions and facilitated later analyses of synergy between elements.

To prevent overfitting, stratified K-fold cross-validation (K = 5 or the maximum feasible value given class sizes) was performed within each gene. Predicted probabilities for held-out reads were collected to compute out-of-fold (OOF) receiver operating characteristic area under the curve (ROC-AUC) and average precision (AP) scores. Because class imbalance was common, AUC was used as the principal, threshold-independent performance metric. A within-gene permutation null was generated by shuffling transcriptional-state labels across reads 50 times and recomputing OOF AUCs. The difference between the observed and permutation-mean AUC (ΔAUC) quantified predictive separation beyond random expectation.

For each gene and transcriptional state, the fitted L1 coefficients were aligned to the full feature set to generate a feature × state matrix of weights. Positive coefficients indicated features whose accessibility increased the probability of the corresponding transcriptional state on the same molecule; negative coefficients indicated a negative association. Coefficient magnitude reflected predictive contribution but not causal effect. Features retained after regularization were used in subsequent analyses of feature importance and regulatory synergy by comparing model performance (AUC) after selective feature removal.

### CRE Dropout analyses

Feature dropout analyses were performed to quantify the contribution of individual CREs to transcriptional-state prediction. This procedure measured the change in cross-validated model performance when one predictor features was omitted. The performance change for each feature was computed as:

$$\Delta A\cup C_{j}=A\cup C\_\text{full}\;-AUC\_{\text{dropout}}_{j}$$

Positive ΔAUC values indicated that a feature contributed to predictive performance, with magnitude reflecting relative importance within that gene and state.

### Synergy between CREs

To test for cooperative effects, models were refit after simultaneous removal of feature subsets S of size k (typically k = 2–4). Synergy was quantified as:

$$\text{Synergy(S)}\;=\left(\Sigma \;\Delta {AUC}_{i}\right)-\Delta AUC\_S$$

Negative synergy values indicated that the joint performance drop from removing all features in S exceeded the sum of the individual drops, implying cooperative rather than additive effects in predicting the transcriptional state.

All dropout and synergy AUCs were obtained using stratified K-fold cross-validation (K = 5 or smaller when limited by class counts) to ensure that ΔAUC values reflected out-of-fold performance differences rather than overfit training improvements. Significance was assessed by repeating the analysis on label-permuted data (N = 50 permutations per gene/state) to generate an empirical null distribution.

### smFISH Probe Synthesis

Probes were designed computationally with a 25-nucleotide hybridization region with a melting temperature of at least 45 C, and specific 20-nucleotide forward primer and 30-nucleotide reverse primer with an incorporated T7 promoter. Probe synthesis followed a modified version of the protocol that was outlined in Mateo *et al. Nature Protocols* 2021 (39).

Briefly, probes were ordered using an oligo library from IDT at 10 pmol/oligo concentration. Probes were amplified with PCR and then treated with a HiScribe T7 in-vitro-transcription (IVT) utilizing the incorporated T7 promoter at 37 C overnight. A specific ATTO-conjugated primer (also from IDT) was added and incorporated into the probes following a reverse transcription reaction with the Maxima H Minus RT kit at 55 C for 5 hours. Resulting ssDNA probes were treated with RNAse A for 0.5 hours at 37 C to remove leftover ssRNA and stored at 4 C in the dark. Intermediary and final products were cleaned with a Zymo RCC-25 kit and reconstituted in nuclease-free H20 following each step.

### smFISH

Single-molecule FISH (smFISH) was performed on embryos fixed in 4% formaldehyde and stored in methanol at – 20 C. We performed smFISH utilizing ATTO565 and ATTO633 conjugated probes. Embryos were washed triple-washed in 1x PBS + 0.1% Tween 20, and then incubated in 35% formamide, 0.1% Tween-20, and 4x SSC for 1 hour at room temperature. Embryos were then hybridized with 750 ng of fluorophore-conjugated probe diluted in 5X SSC Buffer, 50% formaldehyde, 0.1% Tween-20, 10% dextran sulfate, and 100 μg/ml herring sperm DNA at 37 C overnight. The following day, embryos were triple washed in 35% formamide, 0.1% Tween-20, and 4x SSC over a period of 2.5 hours, triple washed and incubated in 1x PBS + 0.1% Tween 20 over 30 minutes and mounted in Prolong Diamond (Invitrogen) to cure overnight.

### smFISH imaging

The embryos were imaged using a Zeiss LSM 880 confocal microscope (Zen software 2.3 SP1) with a Plan-Apochromat ×40/1.3 N.A. oil-immersion objective. Lasers at 488 nm, 561 nm, and 633 nm were used to excite the fluorophores (Alexa 488, ATTO 565, ATTO633). Volumetric field of view was typically set at 354.2 μm × 354.2 μm × 7.5 μm, with a voxel size of 86 nm × 86 nm × 250 nm. Imaging was done with 4x averaging with a pixel dwell time of 8 μs.

### Graph-based regulatory trajectory

Accessible elements surrounding the ftz locus, including the core promoter, were defined by FIRE, and accessibility on each read was assigned based on footprinting patterns. Each read spanning the −6 kb to +2 kb region relative to the ftz TSS was represented by a binary vector of element accessibility. Combinations of accessibility present in <1% of fibers (n < 15) were discarded. All possible routes were then enumerated from initial states (promoter open alone) to peak transcriptional activity (all elements open) and to terminal states (R open alone or U3 open alone). States were not permitted to repeat, and each transition involved the change of only one CRE at a time. Routes were ranked by the number of reads supporting each path, with all paths representing 847 of 916 total active reads and the top-ranked route containing 576 reads.

### Motif enrichment

Transcription-factor motifs were identified within regulatory elements using position-frequency matrices for developmental regulators (e.g. for *ftz*: bcd, cad, ftz, run, odd, prd, h, vfl) were retrieved from the JASPAR 2024 CORE database via the jaspardb interface (42). Each motif’s counts matrix was normalized with a pseudocount of 0.8 and converted to a log-odds position weight matrix (PWM). Sequences were scanned base-by-base using a permissive log-odds threshold (≥ 2.0), and per-position scores were combined across all motif variants for each TF by taking the maximum value.

Motifs were further filtered to retain only those overlapping a putative transcription-factor footprint observed on ≥5% of fibers (generally, n = 100), thereby enriching for actively engaged motifs. For each annotated CRE, motif intensities were summed across the interval, producing a per-CRE, per-TF motif-enrichment matrix. For genome-wide analyses, a restricted set of activator and repressor motifs was used to enable comparisons in motif balance across loci (activators: bcd, cad, dl, vfl; repressors: hb, Kr, run, odd). Motif balance was calculated as the ratio of minimum to maximum activator and repressor motif scores, with zero representing complete imbalance and 1 representing perfect balance.

## Supplementary Material

Supplement 1
