# Supplementary material for: Comprehensive Single Molecule View of Transcriptional Dynamics in Development
Source: bioRxiv. 2025 Oct 29:2025.10.28.685222. Preprint. [Version 1] doi: 10.1101/2025.10.28.685222 (PMC12636477; doi:10.1101/2025.10.28.685222)

bioRxiv preprint doi: https://doi.org/10.1101/2025.10.28.685222; this version posted October 29, 2025. The copyright holder for this preprint (which was not certified by peer review) is the author/funder, who has granted bioRxiv a license to display the preprint in perpetuity. It is made available under aCC-BY 4.0 International license.

Supplemental Figure Legends

Figure S1: Quantification of transcriptional states

A. (Left) example Fiber-seq reads at the *gt* locus showing promoter-proximal paused Pol II footprints. Footprints are labeled by predicted identity (green, nucleosome; pink, Pol II; grey, unlabeled). (Right) min-max normalized enrichment of Pol II footprints at the promoter proximal paused sites for genes binned into deciles of PRO-seq coverage within the promoter region. Error shading indicates 95% confidence interval from 1000x bootstrapped resamplings.
B. (Left) example Fiber-seq reads at the *gt* locus showing PIC footprints. Footprints are labeled by predicted identity (green, nucleosome; pink, Pol II; grey, unlabeled). (Right) min-max normalized enrichment of PIC footprints a the TSS for genes binned into deciles of PRO-seq coverage within the gene body region. Error shading indicates 95% confidence interval from 1000x bootstrapped resamplings.
C. (Left) example Fiber-seq reads at the *gt* locus showing terminating Pol II footprints. Footprints are labeled by predicted identity (green, nucleosome; pink, Pol II; grey, unlabeled). (Right) min-max normalized enrichment of Pol II footprints within the termination region (polyA site to polyA site +500bp) for genes binned into deciles of PRO-seq coverage within the termination region. Error shading indicates 95% confidence interval from 1000x bootstrapped resamplings.
D. (Top) example *in situ* from the Berkeley *Drosophila* Genome Project showing spatial expression of *cnc*. (Bottom) corresponding Fiber-seq tracks showing PRO-seq, a metaprofile of nucleosome and subnucleosome footprints, and 100 randomly sampled Fiber-seq reads at the locus. Footprints are labeled by predicted identity (green, nucleosome; pink, Pol II; grey, unlabeled).
E. (Top) example *in situ* from the Berkeley *Drosophila* Genome Project showing spatial expression of *gt*. (Bottom) corresponding Fiber-seq tracks showing PRO-seq, a metaprofile of nucleosome and subnucleosome footprints, and 100 randomly sampled Fiber-seq reads at the locus. Footprints are labeled by predicted identity (green, nucleosome; pink, Pol II; grey, unlabeled).
F. (Top) example *in situ* from the Berkeley *Drosophila* Genome Project showing spatial expression of *btd*. (Bottom) corresponding Fiber-seq tracks showing PRO-seq, a metaprofile of nucleosome and subnucleosome footprints, and 100 randomly sampled Fiber-seq reads at the locus. Footprints are labeled by predicted identity (green, nucleosome; pink, Pol II; grey, unlabeled).
G. Scatterplot showing correlation of fraction nuclei expressing a gene and fraction accessible chromatin fibers for a subset of 63 highly expressed developmental patterning genes. Histograms on each axis indicate distributions. Pearson correlation is indicated on plot.
H. Scatterplot showing correlation of fraction nuclei expressing a gene and fraction chromatin fibers showing any evidence of transcription (paused Pol II, PIC, elongating Pol II) in Fiber-seq for a subset of 63 highly expressed developmental patterning genes. Histograms on each axis indicate distributions. Pearson correlation is indicated on plot.

bioRxiv preprint doi: https://doi.org/10.1101/2025.10.28.685222; this version posted October 29, 2025. The copyright holder for this preprint (which was not certified by peer review) is the author/funder, who has granted bioRxiv a license to display the preprint in perpetuity. It is made available under aCC-BY 4.0 International license.

I. (Top) mean convoy count and (Bottom) mean convoy size for convoys called using a range of inter-polymerase distance cutoffs. The chosen cutoff (100bp) is indicated with a dashed line.
J. Boxplot comparing distribution of inter-nucleosome and inter-polymerase distances.
K. Min-max normalized enrichment of single or convoy elongating Pol II footprints within gene bodies for genes binned into deciles of PRO-seq coverage within the gene body region.
L. Example Fiber-seq reads from human lymphoblastoid cell lines from the donor for the HG002 assembly within rDNA repeats. Footprints are labeled by predicted identity (green, nucleosome; purple to blue, putative Pol I of 40-80bp size range).
M. Histogram of the ratio of single to convoy Pol II footprints across all genes showing expression in scRNA-seq in NC14.

Figure S2: Extended quantification of hyperburst kinetics

A. Histogram of spacing between elongating Pol II footprints split between single Pol II footprints and convoy Pol II footprints.
N. Lineplot showing count of Pol II footprints within convoys and overall count of nucleosomes within the first 1.5kb of a gene. Error shading indicates 95% confidence interval from 1000x bootstrapped resamplings.
B. Barplot showing fraction of polymerases in different convoy sizes, split into 10 bins of nucleosome eviction rate.
C. Barplot showing the percentage of larger than 2 Pol II convoys in non- and hyperburst fibers. The percent difference is indicated on the plot.
D. Barplot showing the mean count of Pol II convoys per read in non- and hyperburst fibers. The percent difference is indicated on the plot.
E. Barplot showing the Pearson correlation of the 9kb upstream pattern of nucleosome occupancy for active hyperburst fibers to refractory or to non-hyperburst fibers.
F. Scatterplots for the top twelve developmental patterning genes by hyperburst activity. Points represent the percentage of fibers with a Pol II footprints binned by centiles of percent nucleosome eviction.

Figure S3: Prediction of regulatory element function via logistic regression

A. Histogram of the count of transcriptional states predicted (ΔAUC > .05) by the combination of CRE activities observed at a given gene for regression models trained on those combinations on a per-gene basis.
B. Simplex plot showing the balance of logistic regression coefficients for the prediction of five transcriptional states from CRE accessibility for all combinations of two CREs identified at genes with hyperbursting (n= 1250), calculated per-gene. Points are colored based on their strongest correlation coefficient.
C. Simplex plot showing the balance of logistic regression coefficients for the prediction of five transcriptional states from CRE accessibility for all combinations of three CREs identified at genes with hyperbursting (n= 1250), calculated per-gene. Points are colored based on their strongest correlation coefficient.

bioRxiv preprint doi: https://doi.org/10.1101/2025.10.28.685222; this version posted October 29, 2025. The copyright holder for this preprint (which was not certified by peer review) is the author/funder, who has granted bioRxiv a license to display the preprint in perpetuity. It is made available under aCC-BY 4.0 International license.

D. Fraction of genes where each individual transcriptional state was predictable (ΔAUC > .05) by the combination of CRE activities observed at a given gene for regression models trained on those combinations on a per-gene basis.
E. (Top) Histogram of per-gene ΔAUC for promoter accessibility. Median is indicated on the plot. (Bottom) Scatter plot of per-CRE AUC and ΔAUC for promoter accessibility. Density is indicated via colormap.
F. (Top) Histogram of per-gene ΔAUC for pausing. Median is indicated on the plot. (Bottom) Scatter plot of per-CRE AUC and ΔAUC for pausing. Density is indicated via colormap.
G. (Top) Histogram of per-gene ΔAUC for non-hyperburst elongation. Median is indicated on the plot. (Bottom) Scatter plot of per-CRE AUC and ΔAUC for non-hyperburst elongation. Density is indicated via colormap.
H. (Top) Histogram of per-gene ΔAUC for hyperburst. Median is indicated on the plot. (Bottom) Scatter plot of per-CRE AUC and ΔAUC for hyperburst. Density is indicated via colormap.
I. (Top) Histogram of per-gene ΔAUC for termination. Median is indicated on the plot. (Bottom) Scatter plot of per-CRE AUC and ΔAUC for termination. Density is indicated via colormap.
J. Percent gene-body accessibility for combinations of hyperburst-associated CREs, sorted identically to Fig. 3E. Combinations of elements are schematized below, with black boxes showing accessibility. Bars are colored by count of elements accessible. Errorbars represent the 95% confidence interval from 10,000x bootstrapped resamplings of the fibers.
K. Barplot showing the percentage of genes with more than one CRE primarily predictive of hyperbursting, defined as their maximum regression coefficient being the hyperburst coefficient.

Figure S4: Reconstruction of the regulatory trajectory of *ftz* from Fiber-seq reads

A. Heatmap showing logistic regression coefficients for the five CREs identified by FIRE. Coefficients are listed within cells of the heatmap.
B. 100 sampled footprinted Fiber-seq reads at the *ftz* locus where the R element is accessible. Footprints are labeled by predicted identity (green, nucleosome; pink, Pol II; grey, unlabeled).
C. PRO-seq signal within the *ftz* gene. Positive track indicates sense transcription, negative track indicates antisense transcription. The region covered by the R element is schematized on the plot.
D. Representative smFISH image of R-element asRNA localization. Arrows are added to highlight nuclei showing R-element asRNA activity.
E. Enrichment of motifs for activators (top) and repressors (bottom) at the R element, with each submodule indicated via colored overlays. Motifs are filtered based on a permissive log-odds threshold (≥ 2.0), and support of overlapping subnucleosomal footprints in Fiber-seq in at least 5% of total fibers.
F. Graph showing the top route (based on overall count of reads included) for trajectory of transcription activation and attenuation of the *ftz* gene based on Fiber-

bioRxiv preprint doi: https://doi.org/10.1101/2025.10.28.685222; this version posted October 29, 2025. The copyright holder for this preprint (which was not certified by peer review) is the author/funder, who has granted bioRxiv a license to display the preprint in perpetuity. It is made available under aCC-BY 4.0 International license.

seq. Each node represents either opening (top 5 steps) or closing (bottom 5 steps) of the indicated element on the x-axis. Groups of transition steps are colored and labeled on the y-axis as described in the results.

G. Graph showing all possible routes (15 total) for trajectory of transcription activation and attenuation of the *ftz* gene based on Fiber-seq. Each node represents either opening (top 5 steps) or closing (bottom 5 steps) of the indicated element on the x-axis. Groups of transition steps are colored and labeled on the y-axis as described in the results. Edges are thickened based on the count of reads across all trajectories supporting the step.

H. Active footprinted Fiber-seq reads at the *ftz* locus during the 6-8hr period. Footprints are labeled by predicted identity. Footprints are labeled by predicted identity (green, nucleosome; pink, Pol II; grey, unlabeled). The neurogenic enhancer and R element are indicated. Reads are sorted to match the 2-4hr trajectory.

bioRxiv preprint doi: https://doi.org/10.1101/2025.10.28.685222; this version posted October 29, 2025. The copyright holder for this preprint (which was not certified by peer review) is the author/funder, who has granted bioRxiv a license to display the preprint in perpetuity. It is made available under aCC-BY 4.0 International license.

Table S1: Probe sequences used for smFISH

Name	Sequence (5'→3')
**ftz intron 001**	CTTCGCGGTGCACGCATGTCATAAGCAAAGAAAAAATGGGTTAGCAGTATTCGCGCGCATTTGaG
**ftz intron 002**	CTTCGCGGTGCACGCATGTCATGAGTTTTATAACTGCGTATTGTTAGTATTCGCGCGCATTTGaG
**ftz intron 003**	CTTCGCGGTGCACGCATGTCACATATTTTCAATATTTTCAAAGTGAGTATTCGCGCGCATTTGaG
**ftz intron 004**	CTTCGCGGTGCACGCATGTCTTTTTAGGGGTTGTTAATCGTGTGTAGTATTCGCGCGCATTTGaG
**ftz terminator antisense 001**	TCGCGATCGGCACGGATTTGCTTACAGCCACAGTCACAGTCATGGTCGACCGTAAACGCGGAATC
**ftz terminator antisense 002**	TCGCGATCGGCACGGATTTGATGGCCTGCTCAATGATTACCCTCATCGACCGTAAACGCGGAATC
**ftz terminator antisense 003**	TCGCGATCGGCACGGATTTGAGCAGACCCACCAGCAGTACGATGCTCGACCGTAAACGCGGAATC
**ftz terminator antisense 004**	TCGCGATCGGCACGGATTTGACCCGCAGCAGTACCAACATCAGTGTCGACCGTAAACGCGGAATC
**ftz terminator antisense 005**	TCGCGATCGGCACGGATTTGGCTACCAGCAACATCCACAGGACCTTCGACCGTAAACGCGGAATC
**ftz terminator antisense 006**	TCGCGATCGGCACGGATTTGACCATCTGTCTTGAGGTCCGGCGATTCGACCGTAAACGCGGAATC
**ftz terminator antisense 007**	TCGCGATCGGCACGGATTTGTCAGTTACTCTCTTCCCCAGAGCGGTCGACCGTAAACGCGGAATC
**ftz terminator antisense 008**	TCGCGATCGGCACGGATTTGCCGAAAGCCGTACCGCCACGAAACCTCGACCGTAAACGCGGAATC
**ftz terminator antisense 009**	TCGCGATCGGCACGGATTTGAGCGCACTTCTCTCGACCATTTGTATCGACCGTAAACGCGGAATC
**ftz terminator antisense 010**	TCGCGATCGGCACGGATTTGTGACACGCAAATGACACAGCCGAGATCGACCGTAAACGCGGAATC
**ftz terminator antisense 011**	TCGCGATCGGCACGGATTTGGAAGCTGCGACGCGATGAGTTGCACTCGACCGTAAACGCGGAATC

Figure S1

bioRxiv preprint doi: https://doi.org/10.1101/2025.10.28.685222; this version posted October 29, 2025. The copyright holder for this preprint (which was not certified by peer review) is the author/funder, who has granted bioRxiv a license to display the preprint in perpetuity. It is made available under aCC-BY 4.0 International license.

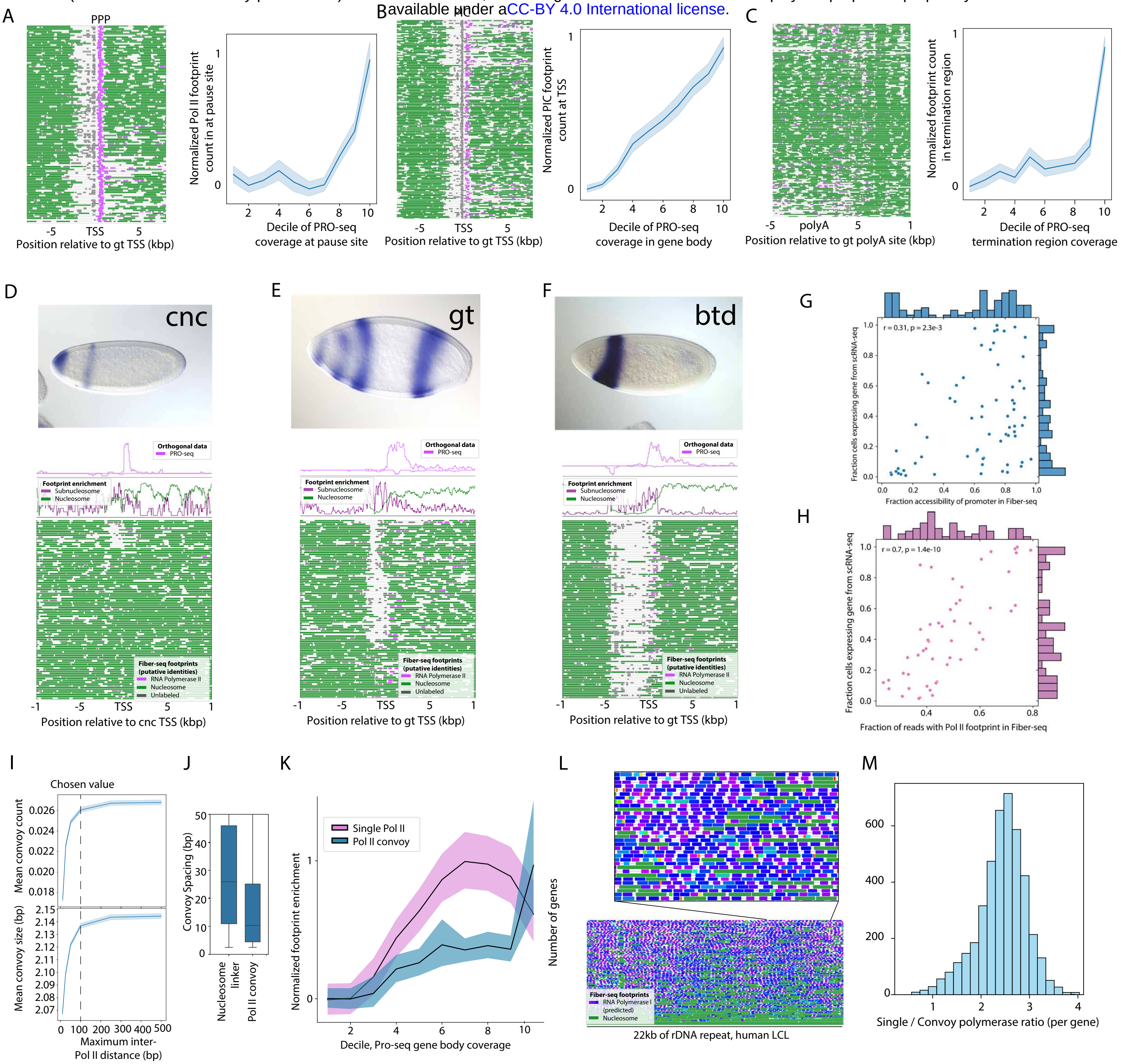

bioRxiv preprint doi: https://doi.org/10.1101/2025.10.28.685222; this version posted October 29, 2025. The copyright holder for this preprint (which was not certified by peer review) is the author/funder, who has granted bioRxiv a license to display the preprint in perpetuity. It is made available under aCC-BY 4.0 International license.

Figure S2

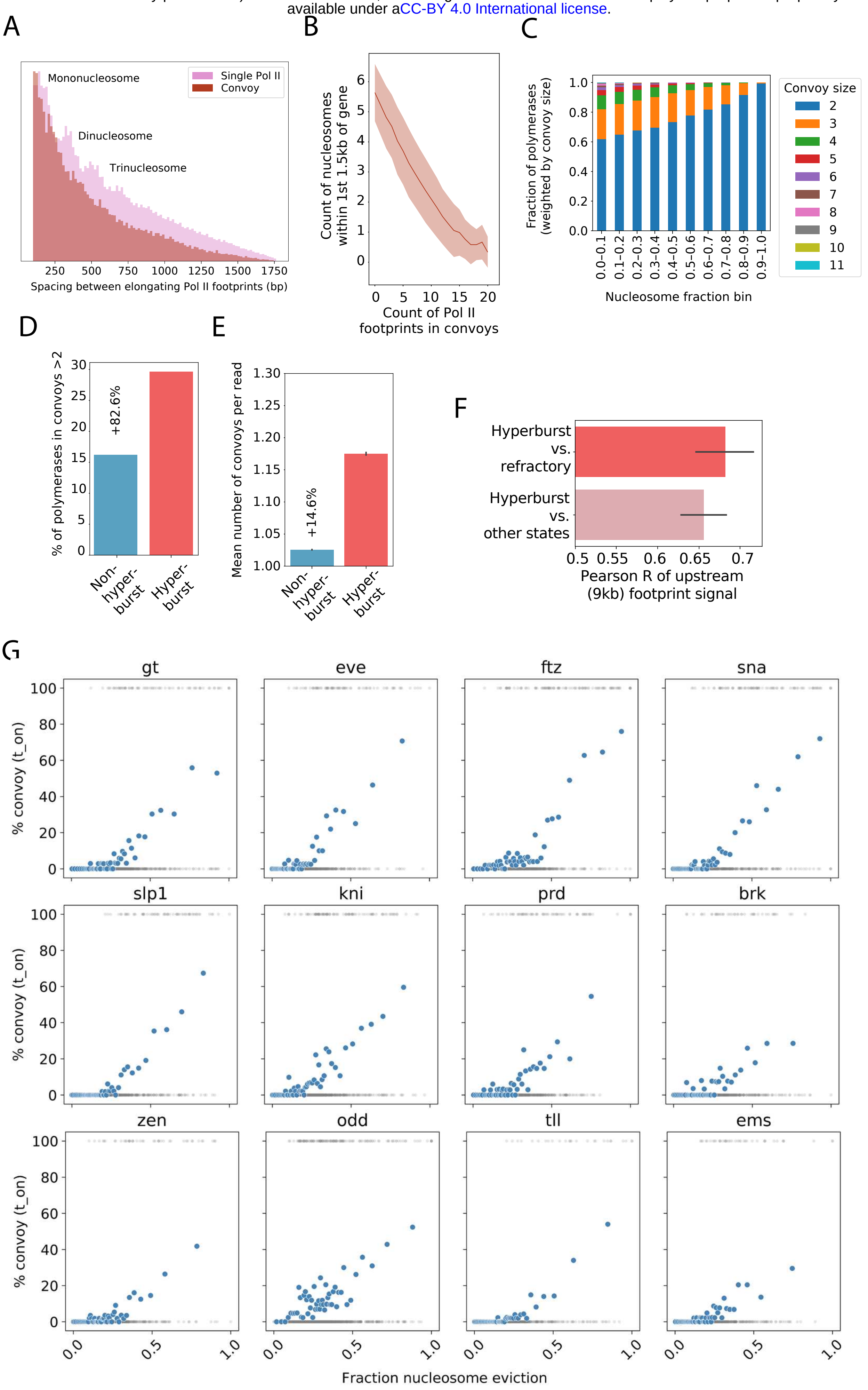

bioRxiv preprint doi: https://doi.org/10.1101/2025.10.28.685222; this version posted October 29, 2025. The copyright holder for this preprint (which was not certified by peer review) is the author/funder, who has granted bioRxiv a license to display the preprint in perpetuity. It is made available under aCC-BY 4.0 International license.

Figure S3

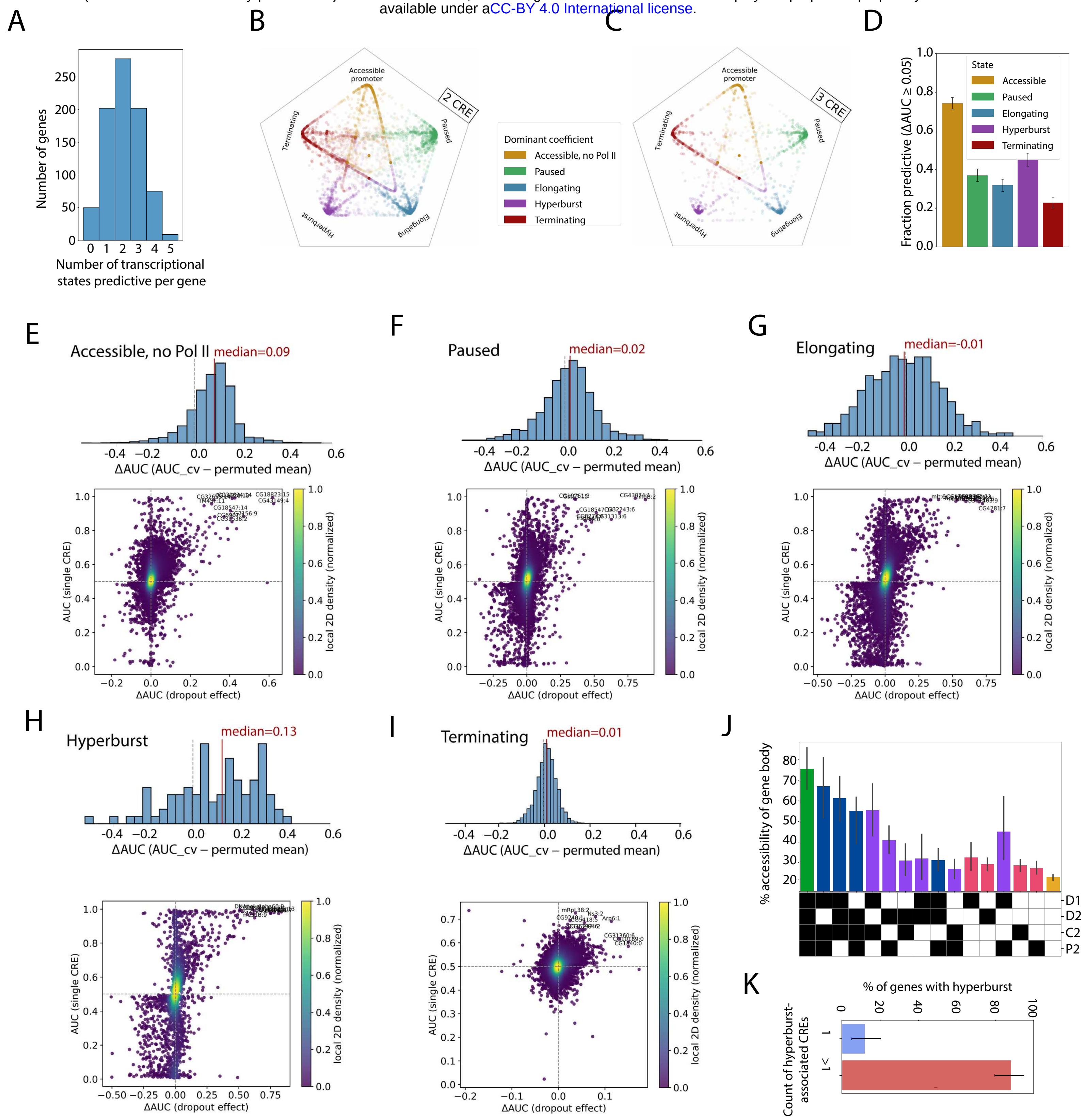

Figure S4 bioRxiv preprint doi: https://doi.org/10.1101/2025.10.28.685222; this version posted October 29, 2025. The copyright holder for this preprint (which was not certified by peer review) is the author/funder, who has granted bioRxiv a license to display the preprint in perpetuity. It is made available under aCC-BY 4.0 International license.

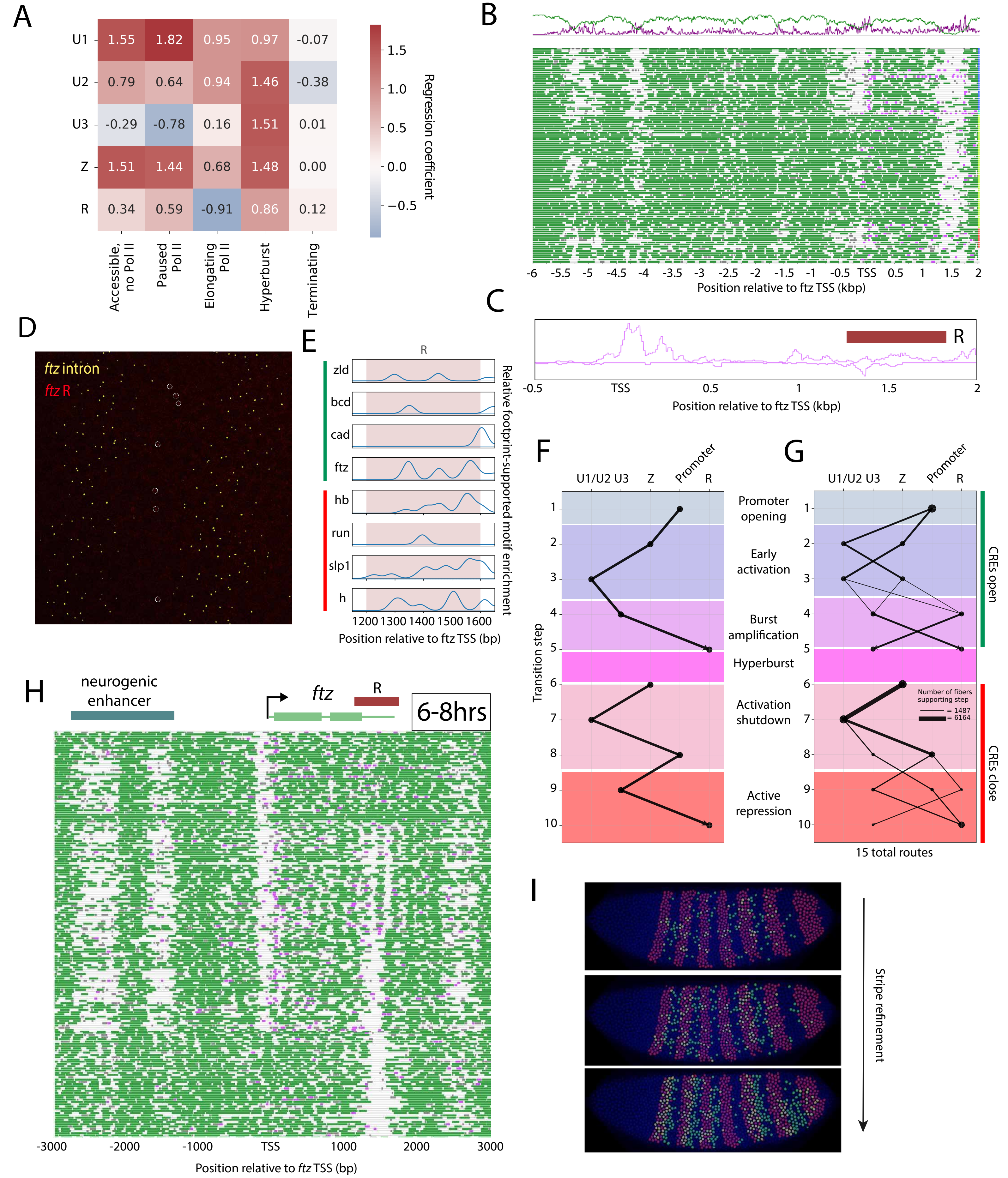

Supplement: Supplement 1 [file NIHPP2025.10.28.685222v1-supplement-1.pdf]
